# Towards accurate high-throughput ligand affinity prediction by exploiting structural ensembles, docking metrics and ligand similarity

**DOI:** 10.1093/bioinformatics/btz538

**Published:** 2019-07-26

**Authors:** Melanie Schneider, Jean-Luc Pons, William Bourguet, Gilles Labesse

**Affiliations:** Centre de Biochimie Structurale, CNRS, INSERM, Univ Montpellier, 34090 Montpellier, France

## Abstract

**Motivation:**

Nowadays, virtual screening (VS) plays a major role in the process of drug development. Nonetheless, an accurate estimation of binding affinities, which is crucial at all stages, is not trivial and may require target-specific fine-tuning. Furthermore, drug design also requires improved predictions for putative secondary targets among which is Estrogen Receptor alpha (ERα).

**Results:**

VS based on combinations of Structure-Based VS (SBVS) and Ligand-Based VS (LBVS) is gaining momentum to improve VS performances. In this study, we propose an integrated approach using ligand docking on multiple structural ensembles to reflect receptor flexibility. Then, we investigate the impact of the two different types of features (structure-based and ligand molecular descriptors) on affinity predictions using a random forest algorithm. We find that ligand-based features have lower predictive power (*r_P_* = 0.69, *R*^2^ = 0.47) than structure-based features (*r_P_* = 0.78, *R*^2^ = 0.60). Their combination maintains high accuracy (*r_P_* = 0.73, *R*^2^ = 0.50) on the internal test set, but it shows superior robustness on external datasets. Further improvement and extending the training dataset to include xenobiotics, leads to a novel high-throughput affinity prediction method for ERα ligands (*r_P_* = 0.85, *R*^2^ = 0.71). The presented prediction tool is provided to the community as a dedicated satellite of the @TOME server in which one can upload a ligand dataset in mol2 format and get ligand docked and affinity predicted.

**Availability and implementation:**

http://edmon.cbs.cnrs.fr.

**Supplementary information:**

Supplementary data are available at *Bioinformatics* online.

## 1 Introduction

Despite the fact that the efforts invested in drug development have constantly increased during the last decades, the number of drug approvals stays almost constant (Munos, 2009). Indeed, about 81% of all new drug candidates fail (DiMasi *et al.*, 2010), mainly due to a lack of drug efficiency and/or side effects associated with off-target binding. In order to reduce time and cost of drug development process, various computer aided methods have been implemented. Two main techniques, namely structure-based and ligand-based virtual screening, are widely used (Lavecchia, 2015; Lionta *et al.*, 2014). They are now routinely used for hit identification in order to prioritize compounds for experimental assays and they are also gaining interest for lead optimization.

**Ligand-based virtual screening** (LBVS) methods are based on analyzing features of substructures and chemical properties related to activity of the ligand. They are useful to search chemical libraries using global or substructure similarity (Mestres and Knegtel, 2000), shape-matching (Nicholls *et al.*, 2010) or pharmacophores (Yang, 2010). The algorithms used in those methods are in constant development and recent LBVS methods are based on data mining and machine learning (Lavecchia, 2015). They do not require structural knowledge of the receptor.

**Structure-based virtual screening** (SBVS) can be used to predict the binding mode of drugs, to define the important specific interactions between ligand and target and finally to discover a way to improve a given drug by guiding further optimization. SBVS includes docking of candidate ligands into a protein target, followed by evaluation of the likelihood of binding in this pose using a scoring function with an important trade-off between speed and accuracy (Cerqueira *et al.*, 2015). Compared to LBVS, which is restricted to similar molecules the training had been performed on, SBVS is applicable to completely new molecules but it requires knowledge of the targeted structure (or reliable theoretical models). Moreover, very small changes or addition of the molecules that can create strong repulsions (e.g. steric clashes) are more likely to be identified by SBVS methods than by LBVS.

**Combining LBVS with SBVS** is emerging as a way to compensate limitations of each of these complementary approaches. Indeed, there are new attempts to combine both, thanks to the increasing number of both atomic structures and affinity measurements. Usually, the combination of LBVS and SBVS is performed in a sequential or parallel manner (Yu *et al.*, 2018; Zhang *et al.*, 2017). The sequential approach uses both methods as filter steps in a hierarchical procedure with increasing refinement. The parallel approach compares the selected compounds of both methods and retrieves either a consensus (selected by both) or a complementary selection (top molecules from each approach) (Lavecchia and Di Giovanni, 2013). Alternatively, one might apply a weak similarity restraint such as a molecular shape restraint for the ligand (to be classified as a shape-matching LBVS method) during the docking process in SBVS as it is implemented in the docking software PLANTS (Korb *et al.*, 2009).

In the present study, we take advantage of a new interface between PLANTS and the web server @TOME (Pons and Labesse, 2009) to screen multiple conformations in parallel (to be described in more details elsewhere). It also allows us to systematically deduce a shape restraint and binding site boundaries based on the geometry of the original ligand from each crystal structure in a fully automatic manner. Subsequent postprocessing is performed using various chemoinformatics tools including several scoring functions to predict protein–ligand affinity and select an optimal pose.

Ultimately, all the parameters computed to evaluate a ligand pose can be used for machine learning. Indeed, the combination of LBVS and SBVS with machine learning is an emerging approach to improve affinity prediction (Wójcikowski *et al.*, 2017). Therefore, we evaluate applicability of machine learning on the docking outputs of @TOME and PLANTS and ligand similarity measurements. In order to set up and evaluate this development, we focused on a well-known therapeutic target—the estrogen receptor ERα.

**The ER**α is a steroid binding receptor playing a key role in a variety of diseases due to its important role in development and physiology. The most prominent examples are ER-based cancer therapies that focus on blocking estrogen action in targeted tissues, with ERα being the main target for treatment of ER-positive breast cancer (Ma *et al.*, 2009). The development of new and improved selective ER modulators is therefore still of high interest for pharmaceutical companies to target tissues selectively and to avoid resistance and adverse effects (Baker and Lathe, 2018; Katzenellenbogen *et al.*, 2018; Wang *et al.*, 2018).

Moreover, ERα can also be an unwanted target of drugs or xenobiotics (Baker and Lathe, 2018; Delfosse *et al.*, 2012) and has been identified as an anti-target that should be considered in toxicity tests during drug development. Thus, a better understanding of the mechanism of ligand recognition by ERα is of paramount importance for safer drug design. Previously, dedicated prediction methods have been addressing the question of whether a molecule is binding or not (Mansouri *et al.*, 2016; Niu *et al.*, 2016; Pinto *et al.*, 2016; Ribay *et al.*, 2016), and traditional structure-activity relationship (QSAR) modeling studies have also been performed with varying success on this nuclear receptor (Asikainen *et al.*, 2004; Hou *et al.*, 2018; Waller *et al.*, 1995; Waller, 2004; Zhang *et al.*, 2013, 2017).

Despite the fact that ERα is an already well characterized therapeutic target (Ekena *et al.*, 1997; Nettles *et al.*, 2004), we are still lacking an efficient and robust method for predicting the binding mode and affinity of docked ligands. A large number of ERα crystal structures in complex with ligands are now known and the binding affinity of hundreds of chemical compounds have been experimentally determined. Therefore, ERα represents a perfect example to attempt a full characterization by combining SBVS with LBVS and employing machine learning in order to better predict binding affinity and potential future drug profiles.

## 2 Approach

Here, we present an integrated approach for high accuracy affinity predictions on the well-known and intensively studied drug target ERα. First, a training set and several testing sets were built by systematic docking of chemical compounds extracted from the BindingDB, the FDA and from in-house experiments, into the available crystal structures of ERα. An interesting feature of the approach is the fact that we take advantage of structural ensembles for the receptor and the ligand to simulate flexibility. Scoring functions and other chemometric information were gathered for the corresponding complexes through the @TOME server and for the ligands through the CDK. All virtual screening results are made available at http://atome4.cbs.cnrs.fr/htbin-post/AT23/MULTI-RUN/FILTER/showform.cgi? WD=AT23/EG/38751543. Seeing that the accuracy of scoring functions is not sufficient for fine ligands ranking, we employ a random forest machine learning algorithm on diverse features, including structure-based docking metrics and ligand-based molecular descriptors. We also tested various subsets of descriptors, such as MACCS fingerprints, and different algorithms. The developed prediction tool is provided to the community as an automatic prediction extension within the @TOME-EDMon server (http://edmon.cbs.cnrs.fr). The developed machine learning method is equally applied on and provided for ERβ and PPARγ.

## 3 Materials and methods

### 3.1 Ligand datasets

#### 3.1.1 BindingDB dataset

Two sets of experimentally tested ligands for the human ERα (UniProtID: P03372) were extracted from BindingDB (Gilson *et al.*, 2016; Liu *et al.*, 2007) (2018 dataset, updated 2018-04-01). One set contains ligands with known inhibitory constant (Ki) as affinity measure, and a second set contains ligands with half maximal inhibitory concentration (IC50) as an affinity proxy.

A few peptides and a series of boron cluster containing molecules were removed from both datasets, as it was not possible to generate proper 3D conformations or charges for these molecules. The final sets contained 281 ligands (Ki set) and 1641 ligands (IC50 set), respectively, with an overlap of 48 common compounds. Overall, both datasets show a similar compound diversity (compare Supplementary Fig. S1). For training, we preferred to focus on the Ki dataset since it corresponds to more direct measurements while the IC50 dataset was used as a larger dataset for method testing.

#### 3.1.2 In-house xenobiotic dataset

The xenobiotic chemical data that was used first as an external testing dataset and afterwards to build an extended training set, is an in-house dataset of 66 ligands with measured affinities for ERα (Grimaldi *et al.*, 2015). These extra compounds correspond mostly to bisphenols, halogenated compounds, as well as phytoestrogens (natural fused heterocycles and macrocycles partially micmicking estradiol).

#### 3.1.3 FDA ERα dataset

In order to have a second external validation, we used the Estrogen Receptor targeted dataset from the Endocrine Disruptor Knowledge Base (EDKB) provided by the U.S. Food & Drug Administration (FDA), named here ER-EDKB dataset. The dataset contains 130 ER binders and 101 non-ER binders including natural ligands and xenochemicals that are structurally different from drug-like molecules. For ER binders, the binding affinity measure is reported as a relative binding activity (RBA), which is based on an assay using rat uteri. Those cell-based measurements are influenced by different factors, such as cellular permeability, and are unfortunately not directly comparable with direct Ki measures. Nevertheless, we predicted affinities using all models and transformed the measured RBA values back to pIC50 values ($\mathrm{pIC}50=\log_{10}(\mathrm{RBA})-8$).

Interestingly, affinity distributions of the datasets cover a wide range of about ten orders of magnitude without major gaps for distinct affinity ranges (see Supplementary Fig. S2).

### 3.2 Generation of ligand conformations

On the ligand side, there are two factors that can have an impact on docking. One is the initial conformation submitted to a docking program. Indeed, providing the bound conformation is a well-known bias to improve the success of a docking tool as previously recognized (Cross *et al.*, 2009; Plewczynski *et al.*, 2011). The generated ligand conformations can also differ significantly due to ambiguities in molecular descriptions (e.g. multiple conformations of heterocyclic alkyl moieties are possible from usual SMILES strings) and to the optimization procedure after *ab initio* building. Indeed, we notice that some steroid compounds highlighted improperly distorted conformation (data not shown). The second factor is the atomic partial charges that have an impact on ligand pose evaluation and can be calculated using different models (e.g. Gasteiger and MMFF94). In PLANTS, the atomic partial charges affect hydrogen bond donor/acceptor properties (e.g. for aromatic carbons) impacting significantly the docking itself and hence its subsequent scoring.

**Fig. 1.**
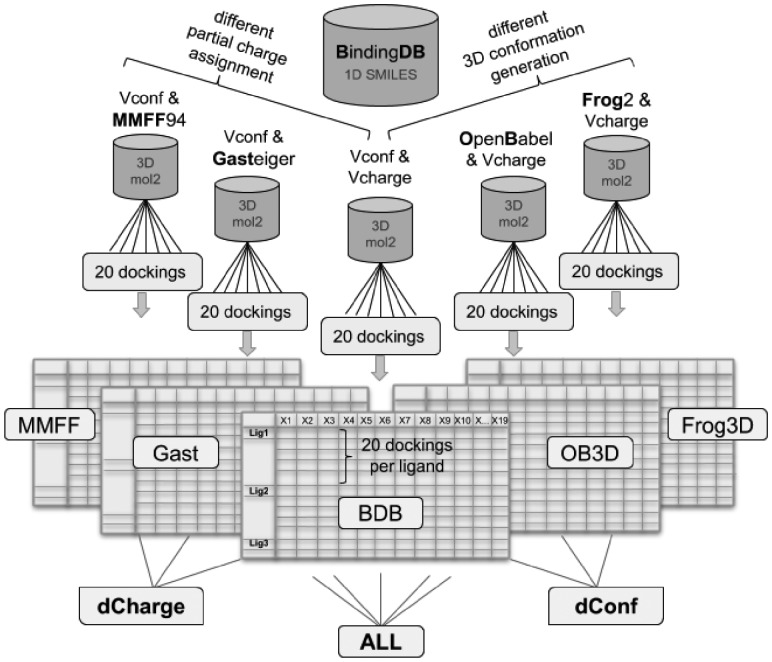
Structure-based dataset generation approach. The ligand dataset was extracted from the BindingDB (BDB), which uses VConf for 3D conformation generation and VCharge for charge assignment. Two more partial charge models (MMFF and Gasteiger) and two other 3D conformation generators (OpenBabel and Frog2) were employed to generate a total of five ligand sets. Those were submitted to the @TOME server for docking and complex evaluation. The @TOME output datasets ‘MMFF’, ‘Gast’, ‘BDB’, ‘OB3D’ and ‘Frog3D’ (containing the results of 20 dockings per ligand in different structures) were grouped in three combined datasets, a different charge dataset ‘dCharge’, a different conformation dataset ‘dConf’, and an ‘ALL’ dataset

The initial ligand sets were downloaded from BindingDB (BDB) and have 3D conformations generated by VConf and partial charges generated by VCharge (Chang and Gilson, 2003). We also tested two other charge models (Gasteiger and MMFF94 charges) instead of the default charge for the 3D conformers built by Vconf. Two other 3D generators [OpenBabel (O’Boyle *et al.*, 2011) and Frog2 (Miteva *et al.*, 2010)] using their default charge. This resulted in a total of five ligand sets. The ligand sets were then grouped based on variation on their 3D generation, their charges or all together as depicted in Figure 1.

Noteworthy, by training on distinctly generated datasets in parallel we prevent dependencies and bring more versatility. Consequently, the user would be able to provide compounds without the need for further conversion that could possibly introduce errors.

### 3.3 Structure-based ligand docking

#### 3.3.1 Ensemble docking

First, all liganded ERα structures available in the PDB (461 monomers) were gathered using the @TOME server (Pons and Labesse, 2009) by submitting the ‘canonical’ amino acid sequence of ERα (UniProt identifier: P03372-1) with a specified sequence identity threshold of 90%. All gathered 461 monomers had a sequence identity between 95 and 100% with the submitted sequence and correspond to point mutants of the human ERα. Missing or substituted side-chains were modeled using SCWRL 3.0 (Wang *et al.*, 2008) using the strictly conserved side-chains fixed. By default, for each ligand to be docked (e.g. from BDB), a set of 20 different template structures were automatically selected among all available PDB structures. This selection is based on the highest similarity (Tanimoto score) between the uploaded ligand and the co-crystallized ligand present in a template. The automatic virtual screening procedure implemented in the @TOME server uses the docking program PLANTS with its shape restraint functionality (with a weighting of -3, the default value suggested by the software manual), using the original ligand of the screened structure as a pharmacophore. Of note, this ligand is also used to define the boundaries of the binding site to be screened (using a distance cutoff of 8 Å). So, not only the protein conformation is (slightly) distinct but various cavity volume and extent are used in this parallel docking procedure. For each template screened, only one pose was computed by PLANTS. After docking and structure alignment, the 20 computed poses were clustered by conformation similarity, and the most likely pose is selected automatically among the largest cluster using a dedicated heuristics. Accordingly, we perform ligand docking on conformational ensemble as an optimal procedure for SBVS.

#### 3.3.2 Structure-based molecular descriptors

Each docking pose is evaluated by various chemoinformatics tools (see Table 1). Here, in order to predict protein–ligand affinities, we take advantage of several re-scoring functions [namely MedusaScore (Yin *et al.*, 2008), DSX (Neudert and Klebe, 2011) or X-SCORE (Wang *et al.*, 2002)) recently embedded in @TOME to derive a consensus score [including also ChemPLP as used in PLANTS (Korb *et al.*, 2006)]. Here, we used both, raw output from these scoring functions, and linear regression based on a study of PDBbind (to be described elsewhere). In addition, other evaluations are performed on the server, such as the model quality of the receptor [QMean (Benkert *et al.*, 2008)] and the evaluation of the ligand conformation [such as LPC (Sobolev *et al.*, 1999) or AMMP energy computed by AMMOS (Pencheva *et al.*, 2008)]. Other scores measure the similarity between the docked ligand and the pharmacophoric anchor used to guide the docking. For instance, AnchorFit (as computed by PLANTS) evaluates their shape similarity, and the Tanimoto score (computed by OpenBabel) evaluates compositional similarity. Finally, we also implemented two new scoring metrics, one comparing ligand–receptor interactions as a sequence-based profile (named PSim for Profile Similarity), and the other predicting a pose RMSD based on a support vector machine (named LPE for Ligand Position Error). These evaluation metrics will be described in more detail elsewhere.

**Table 1.** Structure-based docking metrics

Metric name	Short description
PlantsFull	PLANTS score (with anchor weight) (Korb *et al.*, 2006)
Plants	PLANTS ChemPLP score (without weight)
PlantsLR	PLANTS pKa (calculated by linear regression on PDBbind)
MedusaScore	Medusa original score (Yin *et al.*, 2008)
MedusaLR	MedusaScore pKa (calculated by linear regression on PDBbind)
XScore	XScore affinity score (pKa) (Wang *et al.*, 2002)
DSX	DSX original score (Neudert and Klebe, 2011)
DSXLR	DSX pKa (calculated by linear regression on PDBbind)
AtomeScore	@TOME pKa = mean(PLANTS, XScore, MedusaScore, DSX)
Tanimoto	Similarity between candidate ligand and anchor ligand
AtomSA	S.A. @TOME score
QMean	QMean score of receptor model
AnchKd	Affinity calculated between receptor/anchor (pKa)
AnchorFit	Candidate/ligand superimposition score (PLANTS software)
LigandEnergy	Internal energy of ligand (AMMP force field)
LPC	LPC software score (receptor/ligand complementarity function)
PSim	Similarity to receptor/ligand interaction profile in PDB template
CpxQuality	Complex quality consensus score
LPE	Ligand Position Error (SVM multi-variable linear regression)

The above parameters were important for structure-based screening, and they were complemented by other information regarding the chemical nature of ligands using additional molecular descriptors.

#### 3.3.3 Ligand molecular descriptors

In order to include more information about the small molecules being screened, molecular descriptors were calculated using the Chemistry Development Kit (CDK) (Willighagen *et al.*, 2017), a collection of open source Java libraries for chemoinformatics, through its R interface rcdk (Guha, 2007). The descriptors were selected based on their ability to represent the diversity of the ligand dataset, taking into account their orthogonality, and based on their variable importance score during model training. The final set of 11 QSAR molecular descriptors includes topological, geometrical, constitutional and charge based descriptors and is listed in Table 2 with CDK descriptor name, the used abbreviation and a short description.

**Table 2.** Ligand-based molecular descriptors

Abbrv.	CDK descriptor name	Short description
MW	Weight	molecular weight
VABC	VABC	volume descriptor
nAtom	AtomCount	number of atoms
nBond	BondCount	number of bonds
nRotBond	RotatableBondsCount	number of rotatable bonds
nAromBond	AromaticBondsCount	number of aromatic bonds
nHBDon	HBondDonorCount	number of hydrogen bond donors
nHBAcc	HBondAcceptorCount	number of hydrogen bond acceptors
TPSA	TPSA	Topological Polar Surface Area
XLogP	XLogP	prediction of logP based on the atom-type method called XLogP
HybRatio	HybridizationRatio	fraction of sp3 carbons to sp2 carbons

#### 3.3.4 Combined structure/ligand descriptors

All 5 docking datasets (originating from the 5 different ligand sets) provided 19 structure-based docking metrics for the 20 docking poses computed for each ligand. For each metric, median and standard deviation were computed and used as a unified instance. Ligand-based variables (11 CDK molecular descriptors) were added to the 19 structure-based metrics. A correlation matrix with all descriptors used for the Ki-BDB dataset is provided as heatmap (see Supplementary Fig. S5). Alternatively, the commonly used MACCS fingerprints (166 features) (Durant *et al.*, 2002; Taylor, 2007) were also tested for comparison.

### 3.4 Machine learning approaches

#### 3.4.1 Algorithm selection and training

For all analyses, calculations and machine learning, the R language (version 3.2.4) was used with RStudio (employed packages are listed in Supplementary Table S1). In an initial test on the BDB Ki dataset we assessed the performance of 7 machine learning algorithms (see Supplementary Fig. S3): Random Forest (RF), Gradient Boosting Machine (GBM), support vector machine (SVM) with a radial kernel (SVM_R), a polynomial kernel (SVM_P) and a linear kernel (SVM_L), linear regression (LinReg), decision tree (CARTree) and Partial Least Squares (PLS). All algorithms were employed with default variable settings with the R package ‘caret’. In order to avoid over-fitting of the models, we used stratified 10-fold cross validation repeated 10 times for all models in this study (unless otherwise indicated).

Alternatively, an external test set was built by taking a stratified selection of 20% of the whole dataset. The remaining 80% was used as training set for the models.

#### 3.4.2 Comparison of different tree-based algorithms

The RF algorithm we used, has only one tunable hyperparameter that can be adjusted for the present dataset. Therefore, we wondered whether other tree-based ensemble algorithms with more tunable hyperparameters offer an improved prediction accuracy when tuned more carefully. In total, five different tree-based algorithms were employed on the same Ki BindingDB2018 dataset for affinity prediction and subsequent performance comparison. They are: random forest (RF), regularized random forest (rRF), global regularized random forest (rRFglobal), Extreme Gradient Boosted Trees (xgbTree) and Extreme Gradient Boosted Trees with dropout (xgbDART). Here, Bayesian optimization was employed to select the best hyperparameters (5 to 7 depending on the method), which demands a substantially increase in computational expense compared to the one-variable optimization required for the RF algorithm. The performance of the different models are compared based on the left out data during cross-validation (see Supplementary Fig. S4).

### 3.5 Random Forest regression modeling

Random forest models were trained on each dataset separately (‘MMFF’, ‘Gast’, ‘BDB’, ‘OB3D’ and ‘Frog3D’), on the combination of the three different 3D conformation datasets ({‘BDB’, ‘OB3D’, ‘Frog3D’} = ‘dConf’), on the combination of the three different partial charge datasets ({‘MMFF’, ‘Gast’, ‘BDB’} = ‘dCharge’) and on all five datasets combined (‘ALL’) (compare Fig. 1).

Besides the Pearson correlation (*r_P_*), two further regression evaluation metrics were used to evaluate the model performance on the external test set. First, the coefficient of determination (*R*^2^) is calculated using the sum of squares method. The second metric, the Root Mean Square Error (RMSE) is the average deviation of the predictions (predicted affinities) from the observations (measured affinities). In some cases, we also indicate the Spearman rank correlation (*r_S_*).

## 4 Results and discussion

We developed and tested an automated and integrated structure- and ligand-based approach to predict quickly accurate binding affinities for ERα. This approach takes into account structural variability from the ligand side by using different 3D generators and different charge models, and from the receptor side by using 20 structures for each ligand to be docked. Here, we give access to the docking poses while we evaluate thoroughly the affinity predictions performed using various methods.

### 4.1 Predictions using re-scoring methods

In a first attempt, the predictive power of the four different scoring functions implemented in the @TOME server was assessed.

The Pearson correlations between affinity measurement and the median scores (calculated on 20 docking poses per ligand) are very low for all generated datasets (see Table 3). Even the most recent scoring functions (MedusaScore and DSX) performed poorly in this test. Interestingly, the selection of the best pose among the 20 computed ones slightly improves the correlation between predicted and measured affinities for 3 scoring functions but for MedusaScore which appeared as the most robust and the best for the various ligand description schemes.

**Table 3.** Pearson correlations (*r_P_*) on all five datasets between experimental affinities and scores from four scoring functions Plants, MedusaScore, DSX and XScore, of (1) the best pose selected by @TOME, and of (2) the median scores of the four scoring functions, calculated on 20 dockings per ligand on all five datasets

Dataset name	Plants	MedusaScore	DSX	XScore
(1)	*r_P_* on predictions for the best pose			
Gast	0.042	0.154	0.129	0.060
MMFF	0.063	0.182	0.157	0.082
BDB	0.038	0.111	0.118	0.076
OB3D	0.109	0.180	0.143	0.129
Frog3D	0.022	0.132	0.118	0.040
(2)	*r_P_* on predictions over 20 poses			
Gast	−0.031	0.204	0.019	0.049
MMFF	−0.025	0.192	0.038	0.054
BDB	−0.019	0.087	0.022	0.059
OB3D	−0.017	0.175	0.008	0.050
Frog3D	−0.048	0.199	0.005	0.036

However, the overall correlation is too low for fine ligand affinity prediction and indicates a limitation of the general-purpose scoring functions, but the better Spearman correlation (see Supplementary Table S2) suggested a better ranking that could be useful to guide machine learning. This prompted us to develop a more sophisticated method that should be able to combine advantages of different docking evaluations (structure-based and ligand-based ones) and potentially take into account specific features.

### 4.2 Random Forest regression—model training

First, we did some model optimization using parameter tuning, variable selection and engineering (e.g. to better take into account rotatable bounds, see below).

#### 4.2.1 Structure-based and ligand-based partial models

To investigate the actual affinity prediction capabilities of structure-based and ligand-based variables, partial models were trained using the 19 structure-based metrics or the 11 ligands-based metrics on the same dataset named BDB. Affinity predictions made on the held-out 20% test set are shown in Supplementary Figure S6. The docking-metrics only model (*r_P_* = 0.78, *r_S_* = 0.77 and *R*^2^ = 0.60) outperforms the molecular-descriptors only model (*r_P_* = 0.69, *r_S_* = 0.70 and *R*^2^ = 0.47). Interestingly, the combined model (trained on BDB using simultaneously Vconf and Vcharge data) has Pearson correlation coefficient, Spearman’s rank and *R*^2^ value in between the reduced-variable models (*r_P_* = 0.73, *r_S_* = 0.74 and *R*^2^ = 0.50).

#### 4.2.2 Random Forest model trained on MACCS fingerprints

In this context, it might be interesting to add more information regarding the chemical nature of the ligands studied. Instead of using a reduced set of ligand-based parameters, we turned to use a more thorough description based on an extended and popular fingerprints: MACCS. A new random forest model was trained on MACCS fingerprints representing the ligands only, without providing any structural docking data. This resulted in a Pearson correlation (*r_P_*) of 0.76, Spearman’s coefficient (*r_S_*) of 0.76 and an *R*^2^ of 0.57 on the Ki test set, midway between the two partial models compared above (molecular descriptors only model and docking metrics only model). Combining MACCS with docking-based features improves the overall performance on the training and left-out testing dataset (*r_P_* = 0.81, *r_S_* = 0.82 and *R*^2^ = 0.61) but further evaluation using external datasets suggested some overfitting (see below).

#### 4.2.3 Combined models—trained on single and multiple combined datasets

Then, we compared the various models trained on either single datasets (‘MMFF’, ‘Gast’, ‘BDB’, ‘OB3D’ and ‘Frog3D’) or multiple combined datasets (‘nf’, ‘dCharge’ and ‘ALL’). Whereas the five models trained on single datasets have an *R*^2^ of 0.66 (±0.01), an RMSE of 0.82 (±0.01) and an explained variance of 63.4 (±0.8) during training, the three models trained on multiple datasets have a better *R*^2^ of 0.68 (±0.004), a lower RMSE of 0.78 (±0.008) and an explained variance of 90.6 (±3.7). Also evaluation on the 20% left-out test set demonstrates improved predictions for the models trained on multiple combined datasets (‘dConf’, ‘dCharge’ and ‘ALL’) with a mean Pearson correlation (*r_P_*) of 0.77 (and standard deviation of 0.014), compared to the models trained on single datasets with a mean *r_P_* of 0.73 (and standard deviation of 0.029).

The boosted tree models xgbTree and xgbDART appeared to outperform slightly the RF model on this ‘ALL’ dataset. But the reverse was true when evaluating the corresponding models onto the FDA dataset (see below). Most of the differences are weak and may not be significant. Accordingly, the more complex implementations did not provide significant increase in performance and they were not studied further.

### 4.3 Random Forest regression—model testing

Most remarkable is the strong increase in accuracy when using either the ‘dConf’ model trained on the three different 3D conformation datasets (‘BDB’, ‘OB3D’ and ‘Frog3D’) or the model trained on the fully combined ‘ALL’ dataset comprising all five datasets (‘MMFF’, ‘Gast’, ‘BDB’, ‘OB3D’ and ‘Frog3D’) (compare also Supplementary Fig. S7). Interestingly, using different charge models improves affinity predictions, but slightly less efficiently than using different 3D conformations. This is probably due to the fact that the binding pocket of ERα is mostly hydrophobic and therefore the ligands show the same property and partial charges are predominantly found to differ only marginally.

### 4.4 Analysis of variable importance

**Fig. 2.**
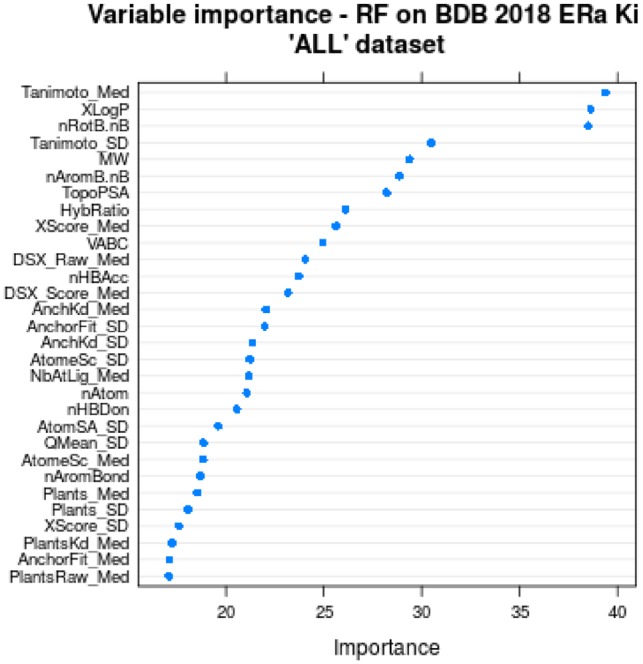
Variable importance of the top 30 variables, tracked during model training for the model trained on the ‘ALL’ dataset with the full variable set. Structure-based docking metrics have an extension (*_Med* or *_SD*). The suffix *_Med* stands for the calculated median of the variable for a ligand’s 20 dockings and *_SD* is the respective standard deviation of this variable

To assess the impact of the various parameters from structure-based and ligand-based scoring functions, the variable importance was tracked during training of the RF models. The 30 most important variables for the models trained on the ‘ALL’ dataset is shown in Figure 2. Overall, all models have a rather similar variable importance profile (data not shown).

Noteworthy, the most important variable *‘Tanimoto_Med’* is the same for all trained models showing its outstanding importance. It represents the median Tanimoto score calculated between the docked ligand and the 20 shape restraints (or ‘anchors’) present in the targeted structure. This may reflect the importance of using structures bound to similar ligand to ensure proper affinity predictions.

**Fig. 3.**
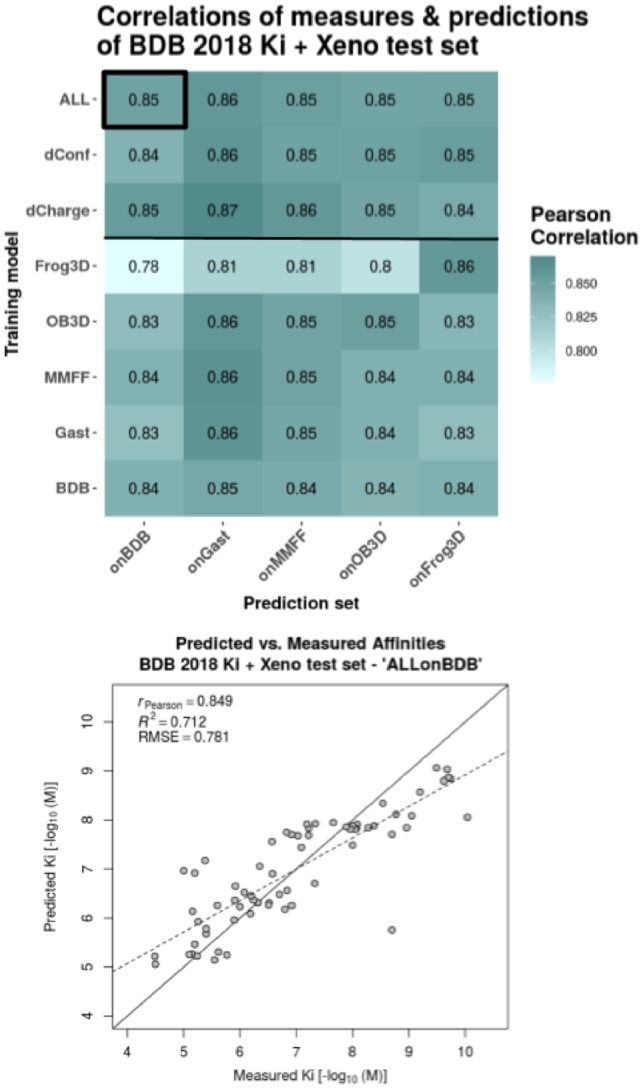
Performance evaluation of extended models on their respective 20% left-out test sets. The initial dataset of 281 ligands is extended by a set of 66 xenochemicals. The heatmap shows Pearson correlations between predictions and measures for all combinations of training model and prediction set. The different training models are listed as rows and the test sets, on which the predictions were made, are listed as columns. RF models were trained on each dataset separately (‘MMFF’, ‘Gast’, ‘BDB’, ‘OB3D’, ‘Frog3D’), on the combination of the three different 3D conformation datasets ({‘BDB’, ‘OB3D’, ‘Frog3D’} = ‘dConf’), on the combination of the three different partial charge datasets ({‘MMFF’, ‘Gast’, ‘BDB’} = ‘dCharge’) and on all five datasets combined (= ‘ALL’). The predictions with the Pearson correlation highlighted in the heatmap (black box) is plotted as scatter-plot for details below. The scatter plot shows the actual predicted versus measured affinities together with a regression line (dashed line), the optimal prediction line (solid diagonal) and the evaluation metrics—Pearson correlation coefficient (*r_P_*), coefficient of determination (*R*^2^) and root-mean-square error (RMSE). All evaluation metrics were calculated with respect to the actual values (solid diagonal), not the regression line

The second and third most important variables are *‘nRotB.nB’* and *‘XLogP’*. *‘nRotB.nB’* estimates ligand flexibility, deduced from the number of rotatable bonds *‘nRotB’* and the total number of bonds *‘nB’* by simply dividing them (*‘nRotB’*/*’nB’*). During variable testing, this combined variable showed an increased importance compared to the original variables (data not shown), which were therefore removed for the final model training. The particular importance of *‘nRotB.nB’* indicates the important role of entropy cost for binding flexible ligands. Obviously, this parameters is not easily handled in a systematic manner by general scoring functions while it is an important parameter for affinity predictions. In the particular case of ERα, it likely discriminates rather small and rigid agonists from larger and more flexible antagonists to prevent overestimating the affinity of the latter. In agreement, the fifth variable is the molecular weight (*‘MW’*) which may also compensate for the additive terms of most scoring functions dedicated to affinity predictions.

Another predominantly important and high-rank variable (second in the ‘ALL’ model and third in the ‘dCharge’ and ‘dConf’ models) is *‘XLogP’*. Representing hydrophobicity and solubility of the ligand, it is expected to be an important factor with respect to the mainly hydrophobic binding pocket of ERα. Moreover, *‘XLogP’* may reflect solvent-driven entropic effects that are not easily taken into account by usual scoring functions. Indeed, flexibility and solvation-linked metrics can be regarded as useful for a crude estimate of some entropic effects and counterbalance the enthalpy-oriented affinity prediction approach of usual scoring functions.

Finally, the different scoring functions (DSX, Plants, MedusaScore and X-score; through their means and standard deviations) show a smaller importance than the three above parameters, which could be in agreement with the poor correlations described above. It may also arise from the intrinsic redundancy of our selected variables as several affinity predictions are used in parallel.

Overall, this result underlines the importance of developing dedicated models for each target under investigation, in order to account for some specific features including particular desolvation and flexibility properties.

### 4.5 Model evaluation on different datasets

#### 4.5.1 Model evaluation on an in-house xenobiotic dataset

We took advantage of a complementary and independent dataset—the xenobiotic chemical data of 66 ERα binders to evaluate the robustness of our models. Our models performed rather poorly on this dataset with a Pearson correlation (*r_P_*) of 0.48 for the best Random Forest model BDB-Ki (and 0.40 with the BDB-Ki+MACCS model). For the partial models, docking-metrics-only and molecular-descriptors-only, as well as the MACCS-only model, correlations are even lower with *r_P_* of 0.45, 0.31 and 0.13, respectively. This underlines the improved robustness of the BDB-Ki model combining SBVS and LBVS features (compare also Supplementary Fig. S8). Importantly, the chemical nature of most xenobiotics differs significantly from most of the drug-like compounds from the BDB dataset used for training. As such, small xenobiotics (including the small bisphenols) occupy only partially the hydrophobic cavity and often also present numerous halogen substitutions (that are notoriously hard to model). Furthermore, for some of the small xenobiotics we cannot rule out the possibility that two molecules may bind simultaneously (with synergetic effects). This result prompted us to combine these xenobiotics and BDB Ki dataset into an extended training set to build a new RF model with improved performance (Fig. 3).

#### 4.5.2 Model evaluation on FDA ER-EDKB dataset

We then evaluated our two best models on a reference dataset comprising both 322 drug-like and xenobiotic compounds (see Table 4 and Supplementary Table S3). At the first glance, the predictions made using the original model (trained on only BDB-Ki) showed a lower performance especially on the edges of the affinity ranges with both overestimated affinities for small and weak binders (e.g. alkylphenol) and underestimated predictions for tight binders such as rigid and compact agonists. Indeed, the BDB dataset is mainly composed of large and high-affinity antagonists. Accordingly, some FDA compounds such as high affinity agonists, appear as strong outliers.

**Table 4.** Model performances on the FDA ER-EDKB test set

Algorithm	Training set	Variable type	Pearson correlation
RF	ALL+Xeno	@TOME+LD	0.748
RF	ALL+Xeno	@TOME+LD+MACCS	0.740
RF	ALL	@TOME+LD	0.663
RF	ALL	@TOME+LD+MACCS	0.648
RF	BDB+Xeno	@TOME+LD	0.712
RF	BDB+Xeno	@TOME+LD+MACCS	0.688
RF	BDB	@TOME+LD	0.584
RF	BDB	@TOME+LD+MACCS	0.542
RF	BDB+Xeno	MACCS only	0.487

*Note*: The presented models employ all the RF algorithm and differ in training set composition concerning used molecules and in type of variables used. @TOME+LD = docking evaluation variables from the @TOME server + ligand descriptors calculated with CDK.

Most remarkable is the benefit of adding a complementary dataset of 66 xenobiotic compounds to the initial 281 ligands from BindingDB (see Table 4). Here, the best Pearson correlation (*r_P_*) of 0.75 is attained with the model trained on ‘ALL’ datasets including the xenobiotics and the model trained on a single dataset (BDB) including the xenobiotics also shows a high *r_P_* of 0.71. Accordingly, the nature and diversity of the ligands matter, so that, proper coverage of the studied chemical space, in the training dataset compared to the testing one is essential. The model trained on a single dataset without the xenobiotics has already a decreased *r_P_* of 0.58, whereas the partial models, docking-metrics-only and molecular-descriptors-only, and the MACCS-only model, show poor performances with *r_P_* of 0.49, 0.47 and 0.41, respectively (compare Supplementary Fig. S9). This underlines again the increased robustness of our feature type combination.

#### 4.5.3 Model evaluation on BindingDB—IC50 dataset

Finally, the most extended and reliable dataset we used for evaluating our RF models is the BindingDB 2018 IC50 dataset which includes 1641 entities. Interestingly, the model trained on the Ki dataset already performed well against IC50 data suggesting a strong robustness.

Training and testing the IC50 dataset (1641 compounds versus 281 for the Ki dataset) also provides some insights into dataset size requirements for the studied target. First, the performance on the IC50 test set (*r_P_* = 0.87) is better than on the Ki dataset (*r_P_* = 0.77) (compare Table 5). Then, cross-predictions were computed by either using the model constructed on the Ki dataset for predictions on the IC50 dataset, or employing the model constructed on the IC50 dataset for predicting the Ki dataset. In that case, it seems that the small Ki test set (56 compounds) does not allow optimal validation as it shows a significant drop in performance compared to the Ki training set (0.49 versus 0.64). On the contrary, the Ki-ALL model showed similar performance on both the IC50 training and testing sets (1319 versus 322 entities).

**Table 5.** Comparison of cross-predictions between the Ki and IC50 models and datasets

	BDB Ki training set	BDB Ki test set	BDB IC50 training set	BDB IC50 test set
number of compounds	225	56	1319	322
Ki ALL model	0.99	0.77	0.64	0.69
IC50 ALL model	0.64	0.49	1.00	0.87

*Note*: Pearson correlations between experimental affinities and the random forest predictions are reported.

We also evaluated our last model trained on the extended dataset including both the Ki dataset and the xenobiotic dataset on the largest available IC50 dataset from BindingDB (compare Table 6). Good predictions were observed for the IC50 dataset although the addition of the xenobiotic dataset did not bring any improvement (nor any deterioration) for that particular dataset. For comparison of all trained model see Supplementary Figure S10. Again, these results suggests that our final model is rather robust.

**Table 6.** Evaluation of best RF models on various datasets

Prediction set	Xeno	FDA	IC50	Ki
RF ALL model				
Ki+Xeno	0.98	0.75	0.65	0.96
Ki	0.48	0.66	0.65	0.77*
IC50	0.25	0.35	0.87*	0.61

*Note*: Pearson correlations between experimental affinities and the RF predictions are reported for the whole datasets but for values marked with ‘*’ that indicates values for a 20% test set.

## 5 Conclusion

We provide an original *in silico* method for accurate binding affinity predictions that takes advantage of structural ensembles, of a limited number of structure-based metrics (19) and of ligand-based descriptors (11) in a unique combination. This combination led to a prediction tool outperforming our other models based either on SBVS or on LBVS features when we take into account not only the overall performance on the internal testing set but also the robustness on a range of distinct datasets. This is true also with the use of many more features as exemplified here with the MACCS fingerprints (166 bits). Our work also confirmed the performance of Random Forest over other machine learning approaches as previously noticed (Russo *et al.*, 2018). In some cases, higher accuracy was reported but for smaller compound libraries (Hou *et al.*, 2018). Accordingly, our results present one of the largest validation surveys (1641 ligands from the BDB IC50 dataset) and best performing tools for affinity prediction against ERα. As major advantages, RF algorithms handle non-linearities, numerical and categorical variables, and they give estimates of variable importance and generalization error.

By training our model in parallel on various types of partial charges and/or 3D builders, we believe our tool will be more versatile and robust to variations in the way the submitted compound libraries are generated. Noteworthy, the user has simply to upload one single dataset to EDMon, where the submitted chemical compounds will automatically be docked and their theoretical affinity for ERα be computed. With this tool, one can easily and rapidly evaluate new compounds either to find putative binders of ERα or to check the absence of binding to this frequent secondary target, in order to avoid potential side-effects.

Interestingly, the same approach yields very similar performances on two other nuclear receptors (ERβ and PPARγ) (see Supplementary Figs S11 and S12, respectively) and their automatic affinity prediction is also implemented in EDMon. The method also provided excellent results for a protein-kinase (to be described elsewhere) and we see no reason for any limitation as soon as dozens or hundreds of structures and affinity points are known. Areas for further improvements are probably: increasing the accuracy in ligand docking, a possible addition of complementary evaluation metrics for the protein–ligand interactions, as well as using deep learning. Testing challenging compounds is also an important way to guide improvement and we expect our web server to be thoroughly tested with novel compounds.

## Supplementary Material

btz538_Supplementary_Data
