# Supplementary material for: Towards accurate high-throughput ligand affinity prediction by exploiting structural ensembles, docking metrics and ligand similarity
Source: Bioinformatics. 2019 Jul 26;36(1):160–8. doi: 10.1093/bioinformatics/btz538 (PMC6956784; doi:10.1093/bioinformatics/btz538)
Supplement: btz538_Supplementary_Data [file btz538_supplementary_data.docx]

Supplement


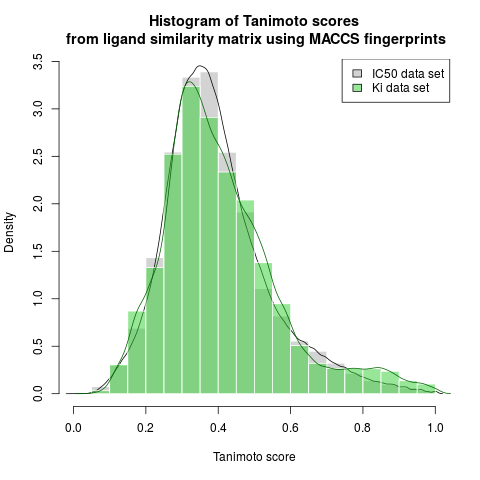


**Figure S1.** Compound diversity of the BDB-IC50 data set and the BDB-Ki data set, evaluated with Tanimoto score on MACCS fingerprints.


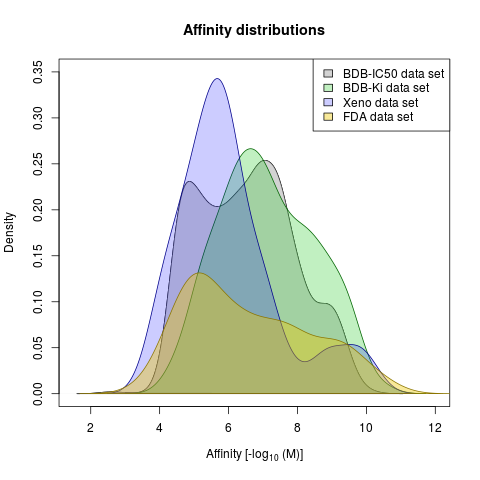


**Figure S2.** Affinity distribution of all 5 data sets: BDB-IC50 data set, BDB-Ki data set, Xeno data set, and FDA data set (with 1641, 281, 66, and 130 compounds, respectively).

**Table S1.** R packages used for computation and visualization


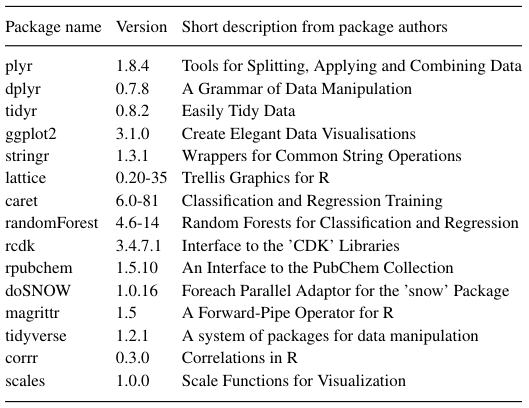


**Table S2.** Spearman’s rank correlation coefficients (r_S_) on all five datasets between experimental affinities and scores from four scoring functions Plants, MedusaScore, DSX and XScore, of (1) the best pose selected by @TOME, and of (2) the median scores of the four scoring functions, calculated on 20 dockings per ligand on all five datasets.


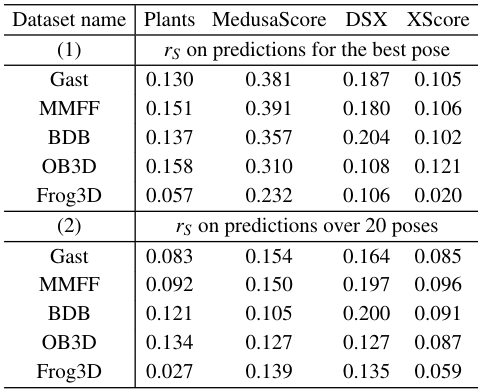


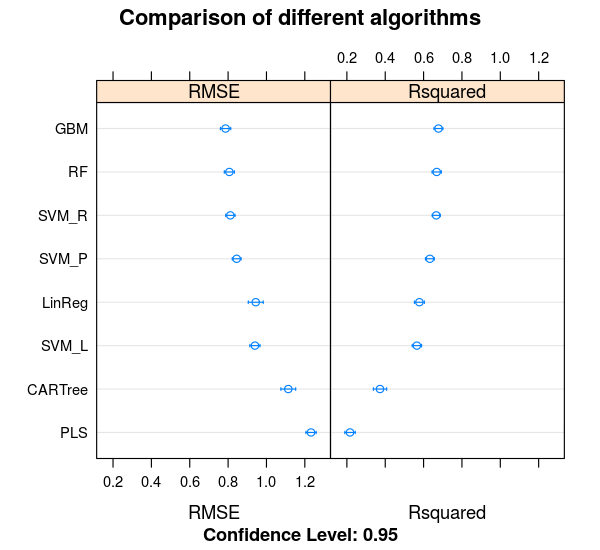


**Figure S3.** Performance comparison of different algorithms for binding affinity prediction (regression) - as dotplot. Models are ranked according to their cross-validation performance: general boosted machine (GBM), random forest (RF), support vector machine with radial kernel (SVM_R), with polynomial kernel (SVM_P), linear regression (LinReg), SVM with linear kernel (SVM_L), Classification and regression tree (CARTree) and Partial Least Squares (PLS).


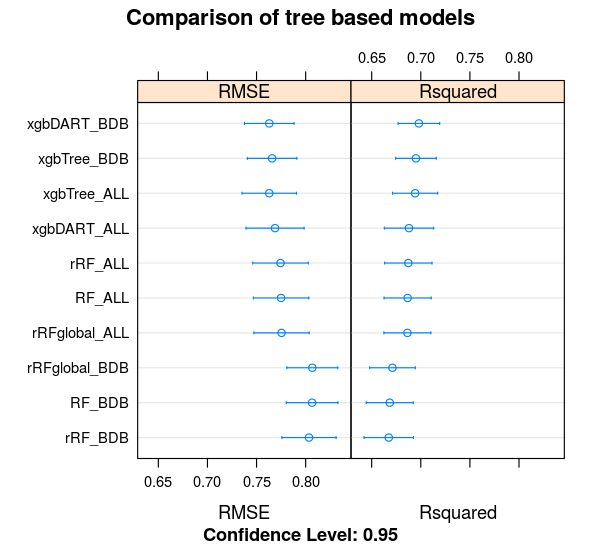


**Figure S4.** Comparison of five different tree-based algorithms with performance metrics calculated on cross-validation samples. All algorithms are trained on the ’BDB’ dataset and on the combined ’ALL’ dataset as indicated by the respective suffix after the algorithm name.


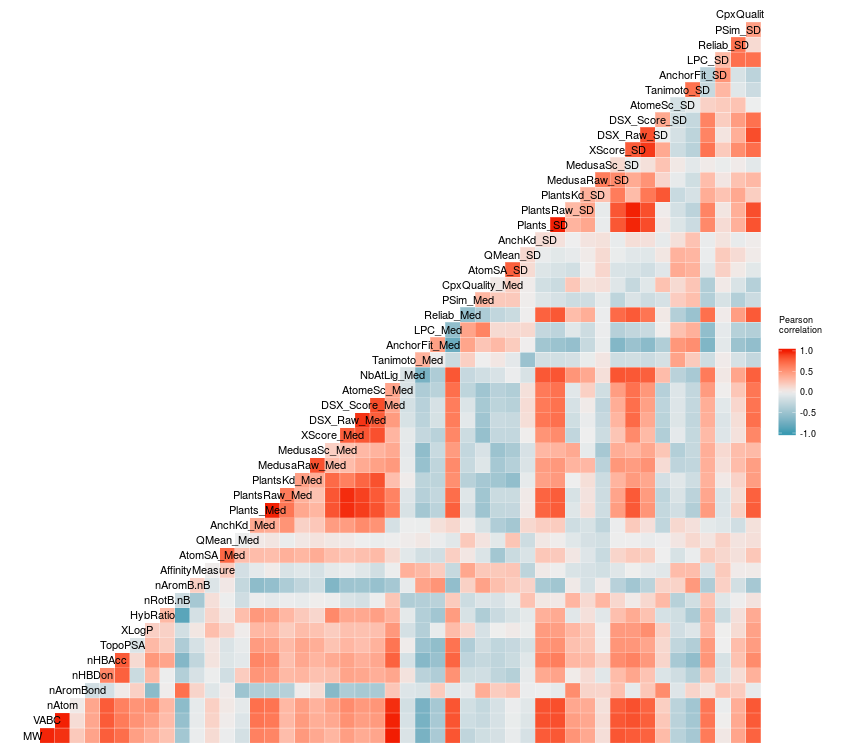


**Figure S5.** Descriptor correlation matrix for the Ki-BDB dataset as heatmap.


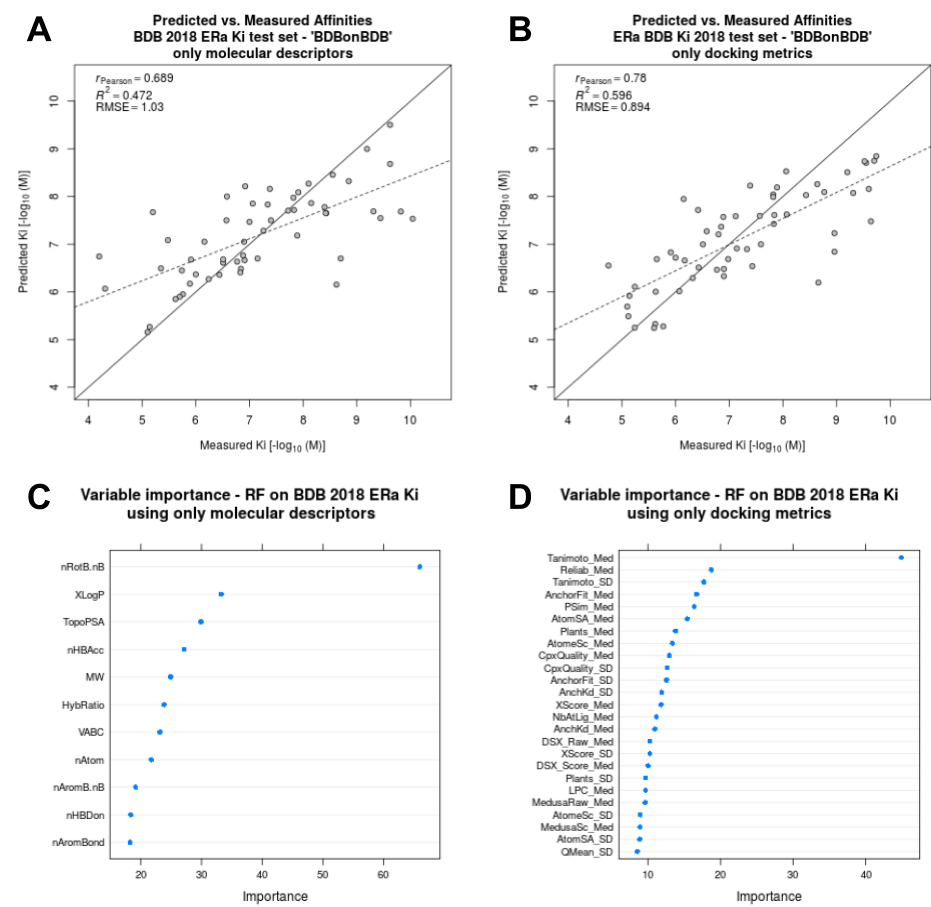


**Figure S6.** Correlations between measured and predicted affinities for the external Ki test set. The predictions were generated by models that were trained on a subset of descriptors. A) model trained only on the set of ligand-based molecular descriptors, and B) model trained only on the set of structure-based metrics from the @TOME server. C) and D) show the ranked variable importance to the trained models A) and B), respectively.


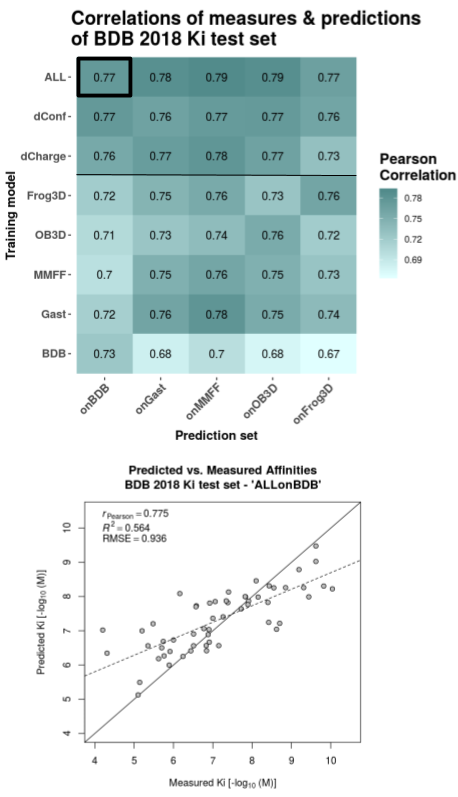


**Figure S7.** Correlations between measured and predicted affinities for the external Ki test set. The heatmap shows Pearson correlations between predictions and measures for all combinations of training model and prediction set. The different training models are listed as rows and the test sets, on which the predictions were made, are listed as columns. Random forest models were trained on each dataset separately (’MMFF’, ’Gast’, ’BDB’, ’OB3D’, ’Frog3D’), on the combination of the 3 different 3D conformation datasets ({’BDB’, ’OB3D’, ’Frog3D’} = ’dConf’), on the combination of the 3 different partial charge datasets ({’MMFF’, ’Gast’, ’BDB’} = ’dCharge’), and on all 5 datasets combined (= ’ALL’). For one prediction set the scatter plot below shows the actual predicted versus measured affinities

together with a regression line (dashed line), the optimal prediction line (solid diagonal) and the evaluation metrics - Pearson correlation coefficient (r_P_), coefficient of determination (R^2^) and root-mean-square error (RMSE). All evaluation metrics were calculated with respect to the actual values (solid diagonal), not the regression line.


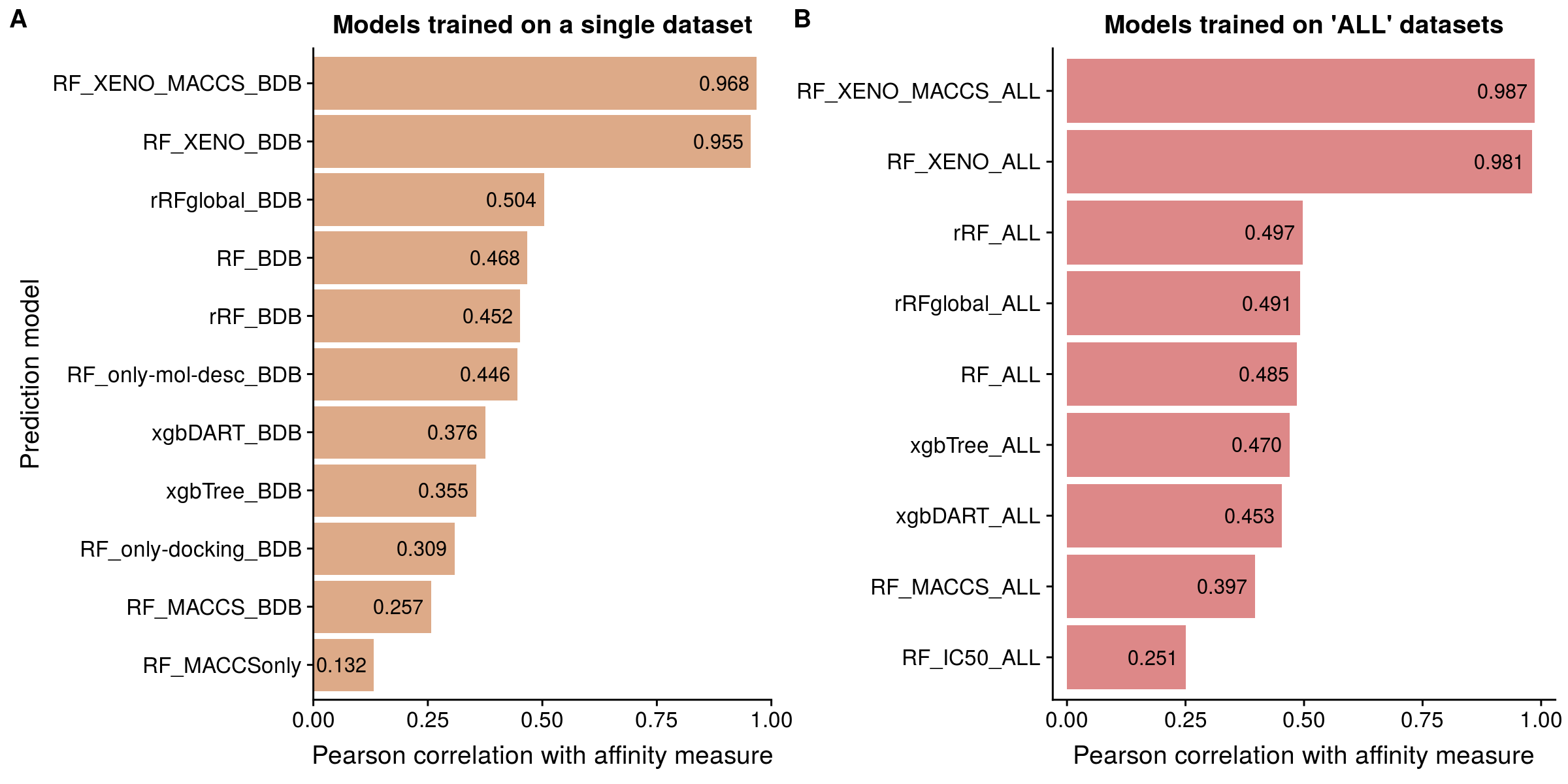


**Figure S8.** Correlations between measured and predicted affinities for the in-house xenobiotic dataset (66 compounds). The prediction models are named by ’algorithm_trainingset’.


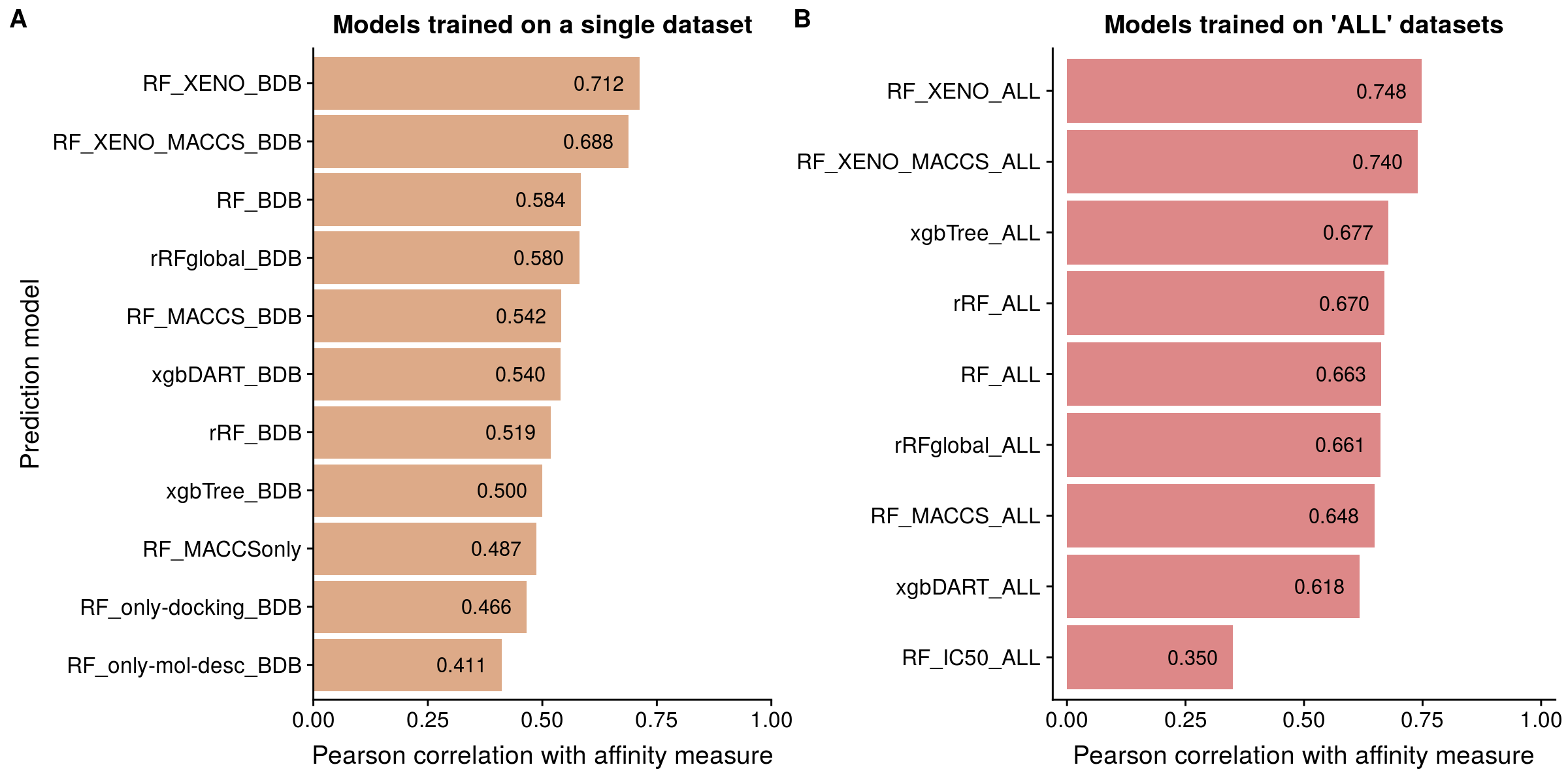


**Figure S9.** Correlations between measured and predicted affinities for the FDA ER-EDKB dataset (131 compounds). The prediction models are named by ’algorithm_trainingset’.


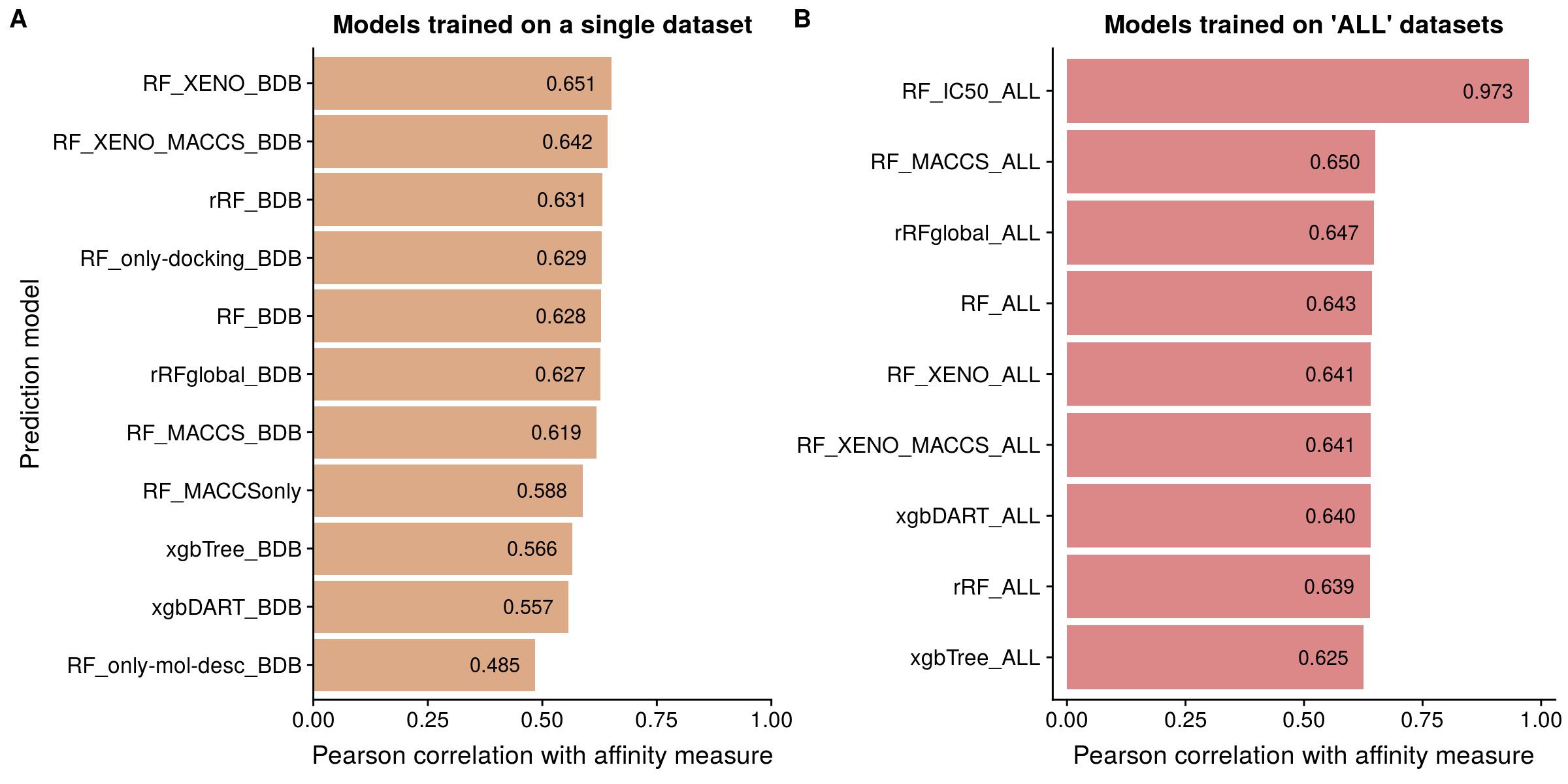


**Figure S10.** Correlations between measured and predicted affinities for the BindingDB-2018 IC50 dataset (1640 compounds). The prediction models are named by ’algorithm_trainingset’.

**Table S3.** Model performances on the FDA ER-EDKB test set. The presented models differ in algorithm usage, amount of cross-validation folds (by default 10-fold unless indicated differently as 3-CV), and training set composition concerning used molecules. The type of variables used remains unchanged. @TOME+LD = docking evaluation variables from the @TOME server + ligand descriptors calculated with CDK.


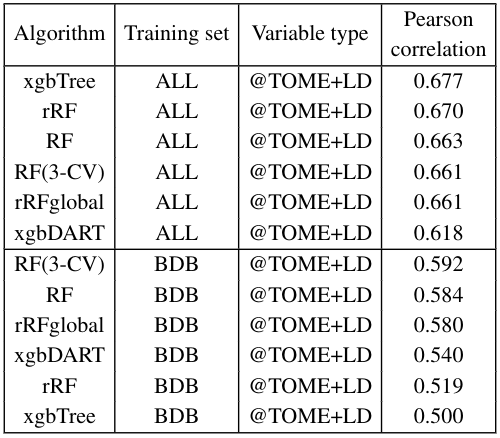


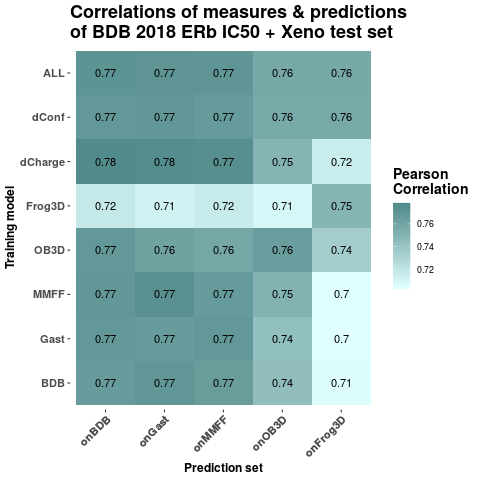


**Figure S11.** Correlations between measured and predicted affinities for the ERβ IC50 + XENO test set. The heatmap shows Pearson correlations between predictions and measures for all combinations of training model and prediction set. The different training models are listed as rows and the test sets, on which the predictions were made, are listed as columns. Random forest models were trained on each dataset separately (’MMFF’, ’Gast’, ’BDB’, ’OB3D’, ’Frog3D’), on the combination of the 3 different 3D conformation datasets ({’BDB’, ’OB3D’, ’Frog3D’} = ’dConf’), on the combination of the 3 different partial charge datasets ({’MMFF’, ’Gast’, ’BDB’} = ’dCharge’), and on all 5 datasets combined (= ’ALL’).


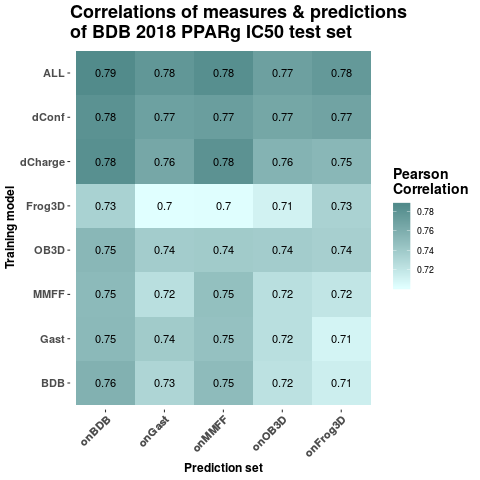


**Figure S12.** Correlations between measured and predicted affinities for the PPARγ IC50 test set. The heatmap shows Pearson correlations between predictions and measures for all combinations of training model and prediction set. The different training models are listed as rows and the test sets, on which the predictions were made, are listed as columns. Random forest models were trained on each dataset separately (’MMFF’, ’Gast’, ’BDB’, ’OB3D’, ’Frog3D’), on the combination of the 3 different 3D conformation datasets ({’BDB’, ’OB3D’, ’Frog3D’} = ’dConf’), on the combination of the 3 different partial charge datasets ({’MMFF’, ’Gast’, ’BDB’} = ’dCharge’), and on all 5 datasets combined (= ’ALL’).
